# Securing China’s chromium supply through circular economy

**DOI:** 10.1016/j.fmre.2025.11.010

**Published:** 2025-11-20

**Authors:** Ziyan Gao, Yong Geng, Wenqiu Cai

**Affiliations:** a School of Environmental Science and Engineering, Shanghai Jiao Tong University, Shanghai 200240, China; b School of International and Public Affairs, Shanghai Jiao Tong University, Shanghai 200030, China

**Keywords:** Critical mineral, Chromium, Supply security, Secondary resources, Scenario analysis

## Abstract

Ensuring a stable and sustainable supply of chromium (Cr) has become an important global issue, as many countries rely on imports to meet their demands and Cr plays a vital role in industrial production. This study aims to address such concerns by tracing the Cr flows and stocks along its life cycle in China for the period of 2020–2023 through a dynamic material flow analysis. Scenario analysis is then conducted to project future Cr supply and demand until 2050 under various circular economy strategies. Results indicate that the total Cr flows and stocks increased slightly from 2020 to 2023 due to the stable growth of stainless-steel industry. The demand for Cr is projected to peak in 2024 as both per capita in-use stock and population approach their maximum levels. Under a combined policy scenario, China’s reliance on Cr imports could decrease to 8% by 2050 if the domestic EoL recycling rate reaches the global leading level (95%) and the primary Cr resource efficiency is improved across all stages of the Cr industrial chain. Furthermore, under a technology breakthrough scenario, demand reduction and material substitution strategies could reduce import reliance to 3% by 2050. These findings highlight the critical role of circular economy strategies, including recycling, substitution, and resource efficiency improvement, in enhancing national Cr supply security. Finally, several policy recommendations are proposed to improve Cr resource efficiency.

## Graphical abstract

**Figure:**
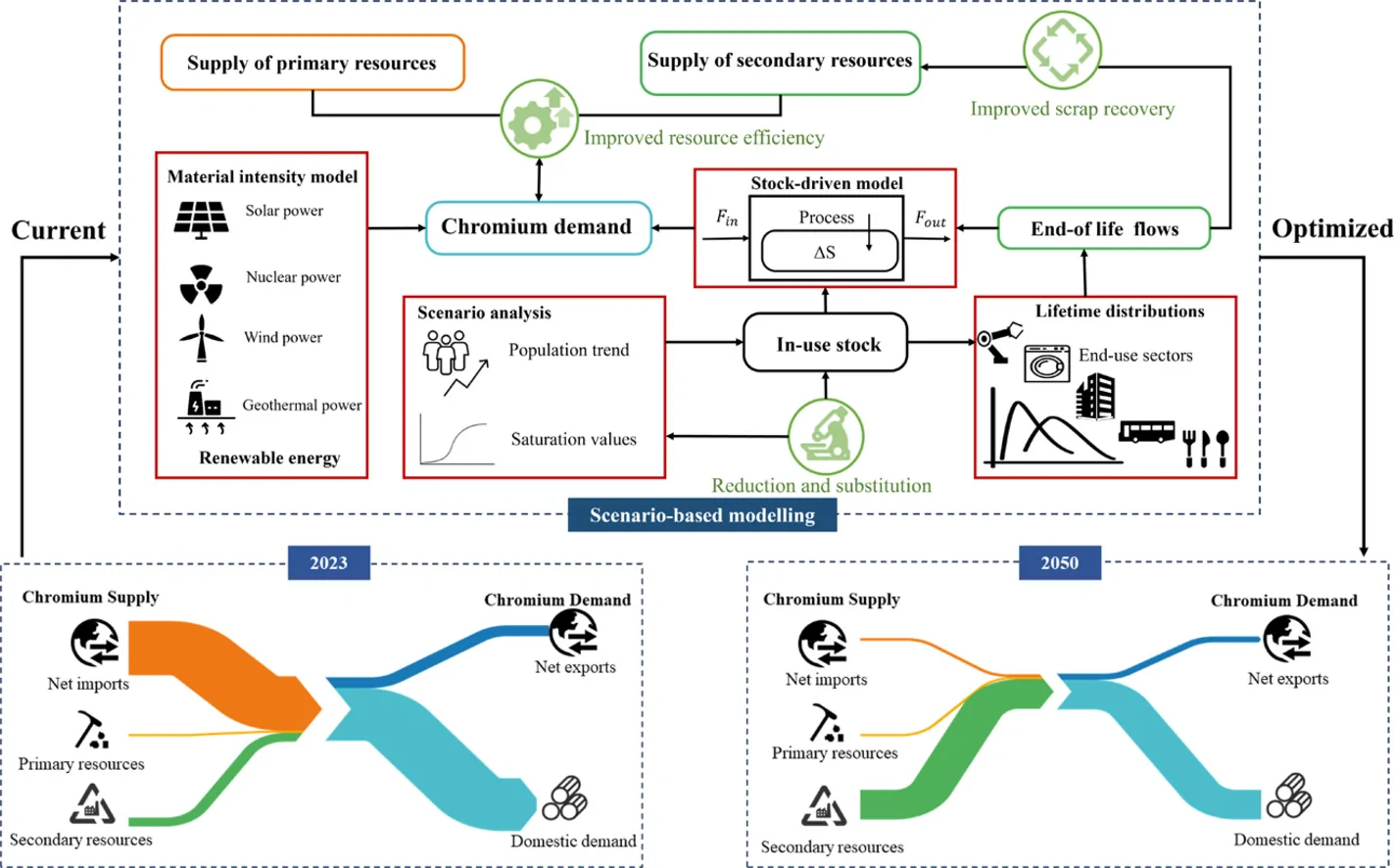

## 1 Introduction

The growing demand for critical minerals and the current geopolitical conflicts have led to global concerns about how to secure a stable supply of such critical minerals [1,2]. Many studies have mainly addressed this issue by focusing on price fluctuations and the supply-demand relation of primary resources [3,4]. But less attention has been paid to the importance of secondary resources. Such efforts are not only essential for achieving Sustainable Development Goal 12 (sustainable consumption and production), but also play a vital role in mitigating supply risks [5,6].

Chromium (Cr) is listed as one of the key critical minerals in many nations due to its irreplaceability in economic development, and the inherent vulnerabilities in its supply chains [7,8]. Stainless steel and alloy steels are the most important Cr applications since it is used to enhance corrosion resistance and strength. These applications accounted for approximately 89% of total Cr consumption in China from 2000 to 2019 (Fig. 1) [9]. Other applications include chemicals, refractory materials, and various chromium-based alloys, which are critical for advanced manufacturing and clean energy technologies [10,11]. The durability, strength, and oxidation resistance provided by Cr-containing materials contribute significantly to the reliability and lifetime of renewable energy infrastructure [12].

**Fig. 1:**
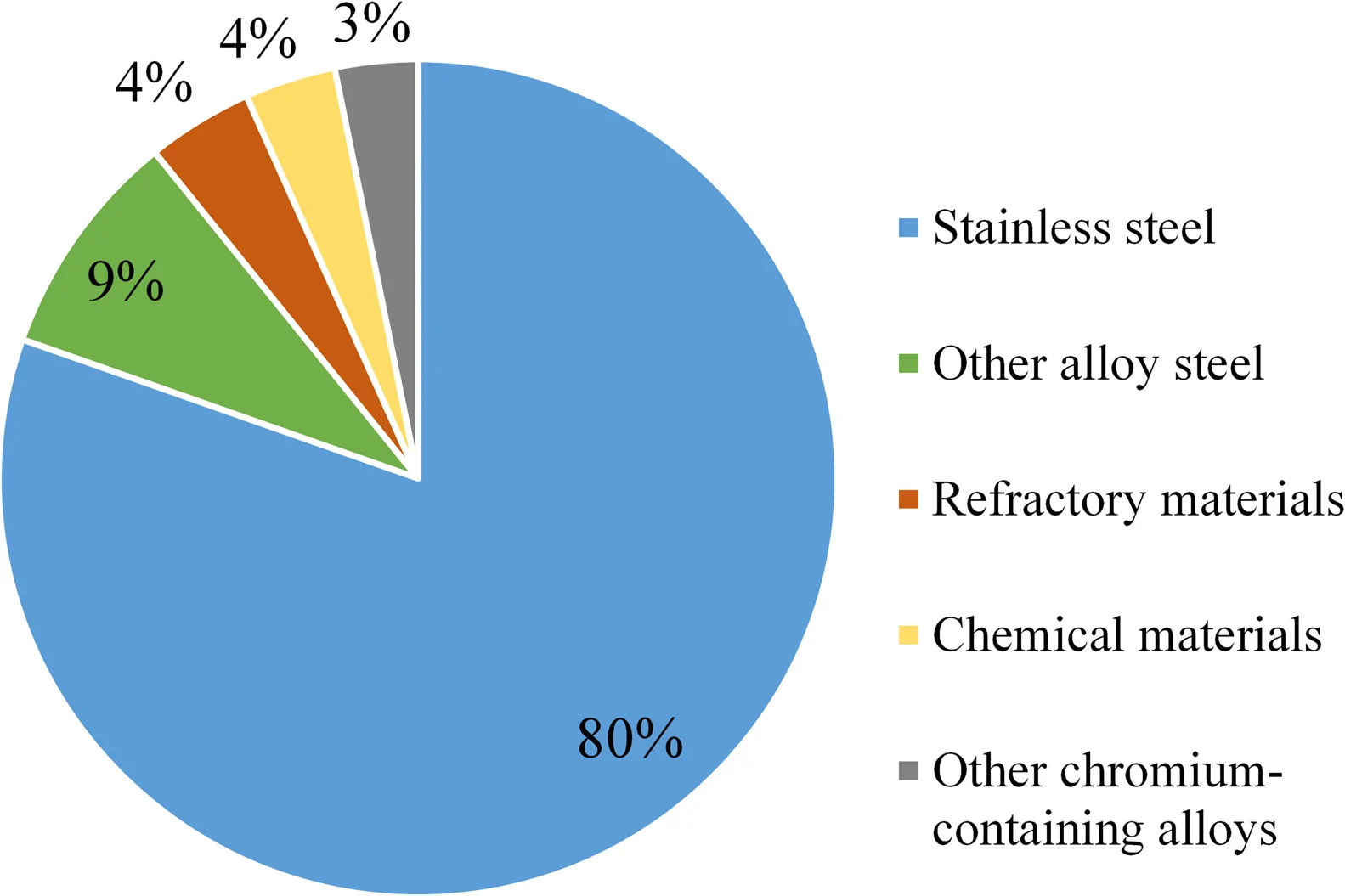
**Sectoral breakdown of chromium consumption in China during 2000–2019 [**9**]**.

In terms of resource endowment, primary Cr resources are mainly concentrated in a few countries, particularly South Africa and Kazakhstan, where political instability and export restrictions pose significant risks to the stable supply of such resources [13]. China is the world’s largest Cr consumer, accounting for over 45% of global Cr consumption and 45% Cr-contained finished products [14]. However, due to limited domestic reserves, it heavily relies on imports from other countries to meet its demand [9,15]. Meanwhile, significant Cr resources are embodied in different products currently in use, indicating a great recycling potential of Cr. Exploring this potential requires a comprehensive understanding of Cr metabolism and accurate predictions on future Cr supply and demand.

To address the sustainability of material supply chains, researchers have developed diverse analytical frameworks, such as sociometabolic models that map material stocks and flows across socioeconomic systems [16,17], and life cycle analysis that assesses environmental impacts from extraction to disposal [18]. Among these methods, material flow analysis (MFA) has been widely adopted to measure the metabolism of critical minerals such as gold [19], gallium [20], germanium [21] and tantalum [22], offering insights into circular economy opportunities for mitigating supply risks. Within this circular economy context, increasing attention has been given to the role of secondary resource supply from end-of-life (EoL) products, especially for materials essential to renewable energy technologies. For instance, Ren et al. [23] projected that by 2050, 26–30% of cumulative metal demand in China’s wind sector could be met through recycling of EoL flows. Similarly, in the solar domain, Rao et al. [24] found that repurposing of EoL photovoltaic panels as a building material can contribute to broader sustainability goals. Complementary studies further highlight that while secondary resources from EoL recycling may play a limited role during the rapid expansion phase of renewable energy, they are critical for ensuring the long-term sustainability of a low-carbon energy system [25,26].

In the case of Cr, MFA studies have been conducted at the national [9,27], global [11,14,28] and product levels [29,30]. Unfortunately, most of these studies focus on the periods before 2020, failing to evaluate the impacts of the COVID-19 pandemic. Recent studies have shown that the COVID-19 pandemic significantly disrupted global resource supply chains by affecting mining operations, delaying international logistics [31], and reducing the diversity of recyclable materials [32]. It is therefore urgent to ensure a sustainable resource supply in the future. Furthermore, existing MFA studies on Cr are largely retrospective. Few of them have projected how circular economy measures [6], such as EoL recycling, resource conservation and demand reduction, might contribute to ensuring the sustainable Cr supply in the future.

In order to address these research gaps, this study pursues two main objectives. First, it aims to uncover the key features of Cr flows and stocks along its life cycle in China from 2020 to 2023 by using the most recent data and employing a dynamic MFA method. This retrospective analysis not only captures recent changes following the COVID-19 pandemic but also provides a critical empirical basis for subsequent projections. Second, based on the historical material flows, a scenarios-based modeling framework is developed to project future Cr supply and demand until 2050, with a focus on evaluating how different circular economy strategies can mitigate import dependence. Together, we expect these efforts to address the Cr supply security issue from a life cycle perspective so that valuable insights can be obtained to support sustainable Cr resource management.

The remainder of this paper is organized as follows: Section 2 details the research methods and data used for tracing Cr flows and stocks, as well as the modeling framework for Cr supply and demand projections. Section 3 presents the research results. Section 4 discusses the uncertainties and limitations of this study and proposes policy recommendations. Finally, Section 5 summarizes the research conclusions of this study.

## 2 Methods and data

### 2.1 System boundary for retrospective material flow analysis

Based on the mass balance principle, MFA is a systematic approach to quantify the flows and stocks of a certain material in a system [33]. It enables a structured assessment of resource use, accumulation and losses across time and space, providing insights into the sustainability and circularity of material systems [5,34]. This study adopts MFA to retrospectively quantify Cr flows and stocks across five key stages of its anthropogenic life cycle in China: production, fabrication, manufacture, use, and waste management (Fig. 2). The overall framework builds upon our previous work on the Cr life cycle [9,14], which established a solid foundation for defining system boundaries and segmenting life cycle stages. The spatial boundary of this study is China’s mainland, while the temporal boundary is from 2020 to 2023, which reflects the very recent features of Cr metabolism. All the flows and stocks are measured in Cr metallic equivalent. The accounting method of such flows and stocks follows the mass balance principle and is shown in Eq. (1):

(1) $$F_{i,j}^{input}+F_{i,j}^{import}+F_{i,j}^{recycling}=F_{i,j}^{output}+F_{i,j}^{export}+F_{i,j}^{loss}+\Delta S_{i,j}$$

where *i* denotes the *i*th life cycle stage, *j* denotes the *j*th year during this study period, $F_{i,j}^{input}$ is the Cr contained in domestic feed-in flows from *i*th life stage in year *j*, $F_{i,j}^{import}$ represents the Cr embedded in imported products from the *i*th life stage in year *j*, $F_{i,j}^{recycling}$ represents the recycled Cr amount in *i*th life stage in year *j*, and $F_{i,j}^{output}$ represents the Cr contained in products consumed by domestic demand from the *i*th life stage in year *j*, $F_{i,j}^{export}$ represents the Cr embedded in exported products in year *j*, $F_{i,j}^{loss}$ is the Cr loss during the *i*th life stage in year *j*, $\Delta S_{i,j}$ is the net Cr addition to the Cr stock in the *i*th life cycle in year *j*.

**Fig. 2:**
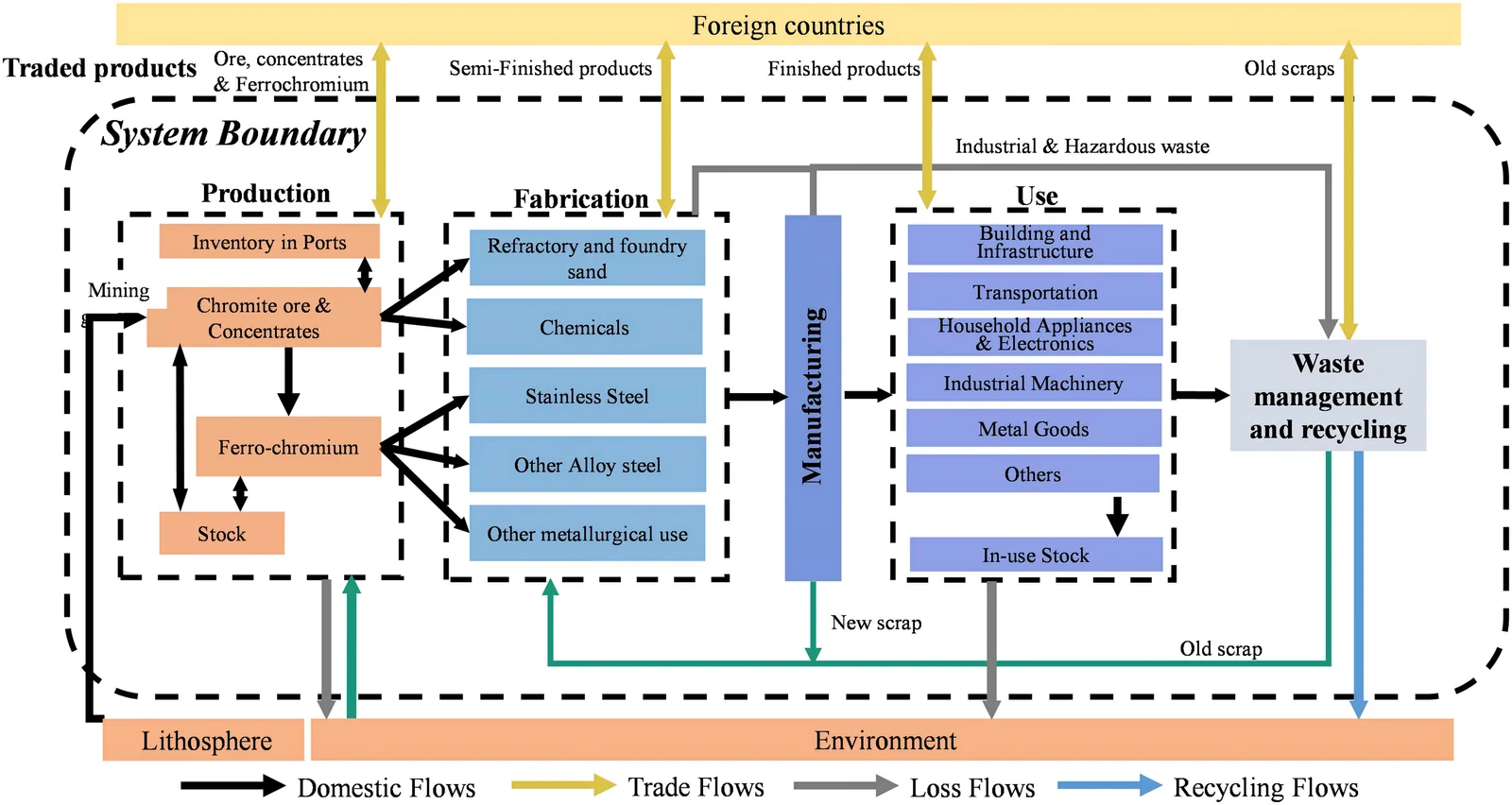
**Material flow analysis framework for the anthropogenic Cr cycle**.

### 2.2 Modeling framework for Cr supply and demand projections

To estimate the future Cr supply and demand in China, a scenarios-based modeling approach is employed. Fig. 3 illustrates this estimation framework. The forecasting period is from 2024 to 2050. Two models are used to project the demand for Cr. The first one is a stock-driven model for the steel sector, and the second one is a material intensity model for the renewable energy sector. As illustrated in Fig. 2, the main Cr application fields include stainless steel, other alloy steels, refractory materials, and chemicals. Specifically, the demand for Cr in those refractory materials closely reflects the trend of the steel industry since these materials are primarily utilized in steelmaking, such as open-hearth furnaces and electric arc furnaces [35]. Due to this close correlation, Cr flows contained in stainless steel, alloy steels, and refractory materials are grouped into this “steel sector” for the purpose of Cr demand projection. The demands for chemical materials and other metallurgical applications, such as aerospace alloys and high-performance superalloys, are not further discussed due to their relatively small contributions to the overall demand.^1^

**Fig. 3:**
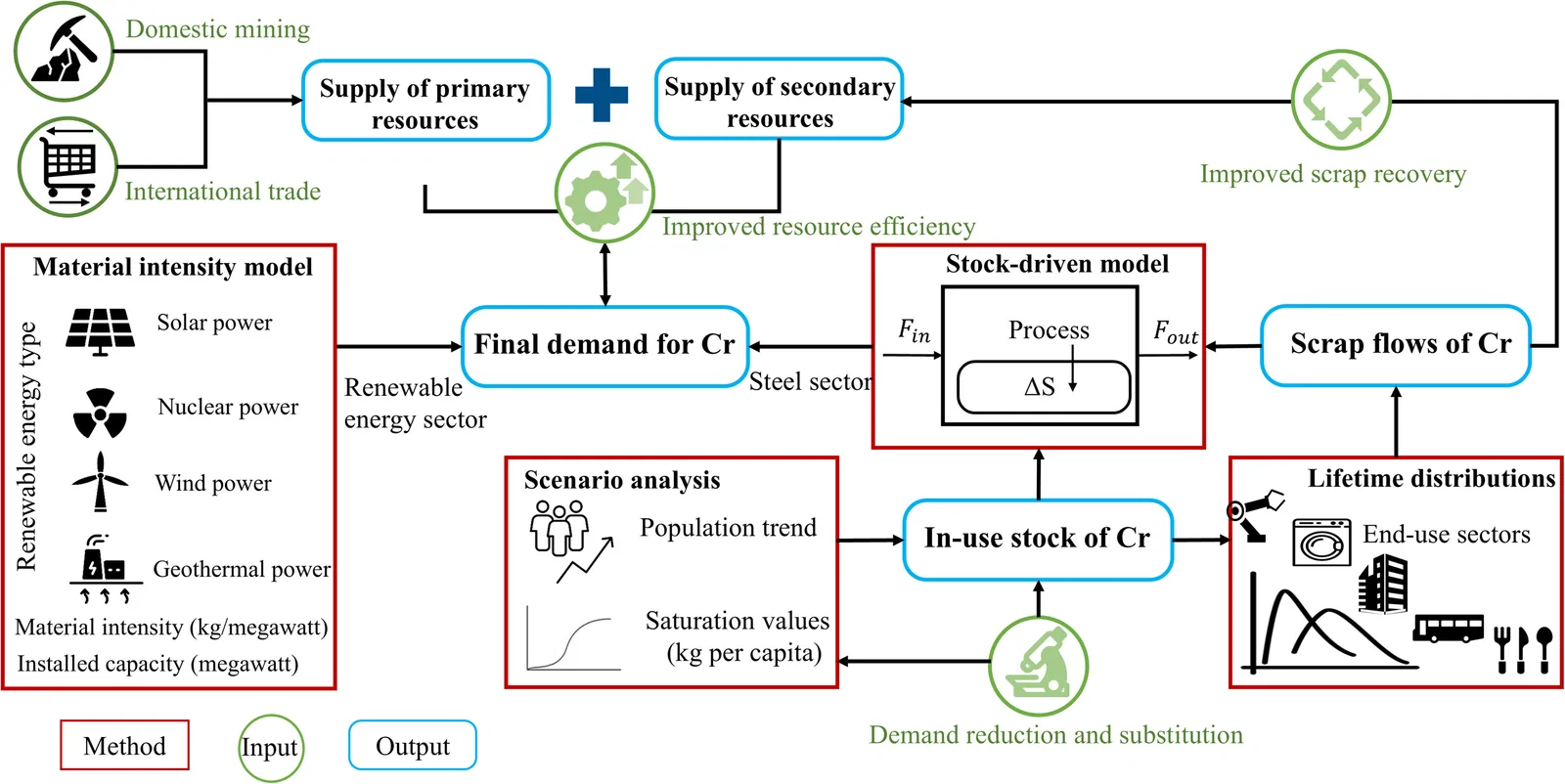
**Modeling framework for projecting future Cr supply and demand**.

Additionally, although renewable energy is included in the “Others” end-use sector in retrospective MFA, it is considered separately in this projection analysis because its technological development and large-scale application have significant impacts on the demands for critical metals [36]. Cr is widely used in the renewable energy sector, including solar panels, thermal storage materials for geothermal energy, structural materials for wind turbines, and nuclear reactors [10]. Thus, this study uses the material intensity of Cr across different renewable energy technologies and the projected installed capacity targets to estimate the future demand for Cr in this sector.

In terms of Cr supply projection, this study developed six scenarios to simulate the potential impacts of different circular economy strategies on the allocation of primary and secondary Cr resources. These strategies include resource conservation, recycling, demand reduction and substitution.

#### 2.2.1 Stock-driven model for the steel sector

Since most steel products typically have longer lifespans (15–20 years), their in-use stocks are relatively stable [37]. This study uses a stock-driven model to estimate the future demand for Cr in the steel sector. This model assumes that such demand is primarily driven by the in-use stock within the socio-economic system. By following the principle of mass conservation, we account the future Cr inflows by using Eq. 2.

(2) $$F_{in,t}=F_{out,t}+S_{t}-S_{t-1}$$

where $F_{in,t\;}$represents the Cr inflow during the use phase in year *t*, $F_{out,t}$ represents the Cr outflow during the use phase in year *t*, which is estimated using a Weibull distribution model calibrated with sector-specific empirical data (see Appendix Table A1), $S_{t}\;$and $S_{t-1}$ represent the in-use Cr stock in year *t* and *t-1* respectively. The trajectory of the future Cr stock is projected by considering the dynamics of per capita stock and population.

The per capita stock of bulk metal resources (such as copper and steel) typically follows an S-shaped growth curve [38,39]. Normally, such a stock first increases slowly and then rapidly increases during the industrialization period and eventually saturates when the local economy reaches a certain level. Such a trend has been observed in the United States, Japan, the United Kingdom, South Korea and Sweden [40,41]. Since over 90% of Cr used in the steel sector is for stainless-steel production, we assume that China’s future per capita Cr stock in the steel sector follows such a trend that industrialized countries experienced [42]. The per capita Cr stock can be calculated by using the logistic equation [43], which is shown in (3), (4), (5).

(3) $$PS\left(t\right)=\frac{PS_{a}}{\left(1+a\;*\exp\left(-r\;*\;PS_{a}\;*\;t\right)\right)}$$

(4) $$a=\frac{PS_{a}}{PS_{0}}-1$$

(5) $$r=\frac{PS_{0}{}^{\prime }}{PS_{0}\;*\;\left(PS_{a}-PS_{0}\right)}$$

where PS(t) represents the per capita Cr stock in year *t*, $PS_{a}$ represents the saturation level of per capita Cr stock, $PS_{0}$ is the per capita stock in the initial year 2023, $PS_{0}{}^{\prime }$ is the initial growth rate of per capita Cr stock in 2023, a is a parameter derived from the initial and saturated stock levels, and r is a constant representing the intrinsic growth rate of the per capita stock

This study designs high, medium and low scenarios to reflect different per capita stock saturation levels for each end-use sector (Table 1). The saturation values are set based on the development patterns observed in those developed countries and the sectoral development plans in China [41,[44], [45], [46], [47]]. The detailed reference basis and numerical data supporting scenario settings are provided in the Appendix.

**Table 1:** **Saturation levels of per capita Cr in-use stock under three scenarios (Unit: kg/person)**.

Sector	Infrastructure	Mechanical equipment	Electronic equipment	Metal products	Transportation	Other uses	Total
Low-saturation scenario (LS)	3.8	5.5	4.0	10.4	3.5	2.8	30.0
Medium-saturation scenario (MS)	4.0	6.0	4.5	10.5	4.0	3.0	32.0
High-saturation scenario (HS)	4.2	6.2	4.7	10.8	4.3	3.3	33.5

#### 2.2.2 Material intensity model for the energy sector

The demands for metals in renewable energy are primarily shaped by material intensity and capacity expansion plans [48]. In this material intensity model, a bottom-up approach is employed to estimate future Cr demand in renewable energy sector, which is shown in the following equation:

(6) $$P_{n,t}=MI_{n}*CA_{n,t}$$

where $P_{n,t}$ represents the demand for Cr in renewable energy type *n* in year *t*, $MI_{n}$ is the material intensity of Cr for renewable energy type *n* (as shown in Table 2), and $CA_{n,t}$ represents the newly installed capacity of renewable energy type *n* in year *t*.

**Table 2:** **Cr material intensities for different renewable energy types** [12,49].

Types of renewable energy	Material intensity	Unit
Solar	6	kg/MW
Wind	400	kg/MW
Nuclear	2190	kg/MW
Geothermal	65,000	kg/MW

This study assumes a consistent growth rate for installed renewable energy capacity within each five-year plan period. Consequently, the annual newly installed capacity is considered unchanged within any given five-year period (Table 3). The target for the total installed power generation capacity from 2021 to 2050 is based on a scenario with a high renewable energy penetration, which aligns with the global vision of advancing renewable energy development and minimizing the use of fossil fuels [50]. In this context, China is committed to maximizing its renewable energy development to achieve its ambitious carbon neutrality targets.

**Table 3:** **Installed capacity for electricity generation in the high proportion renewable energy scenario (Unit: Megawatts)**.

Types of renewable energy	2020	2025	2030	2035	2040	2045	2050
Solar	253,430	500,235	1,048,858	1,657,055	2,206,333	2,678,200	2,696,164
Wind	281,530	632,176	1,103,944	1,663,348	2,092,248	2,287,630	2,396,591
Geothermal	50	1230	2460	3750	11,250	11,250	11,250
Nuclear	49,890	64,000	66,000	69,000	78,000	85,000	100,000

#### 2.2.3 Scenarios development

From a supply chain perspective, several measures can be taken to ensure future Cr resource supply, including resource expansion, conservation, recycling, substitution (including both resource substitution and functional substitution) and circular design [51]. However, China has very limited Cr resources, making it difficult to increase its domestic primary Cr resource extraction [14]. Also, the irreplaceability of Cr in specific applications, such as stainless steel and alloy steels, makes Cr resource substitution impossible under current technological and market conditions [30]. Therefore, this study primarily evaluates the impacts of resource conservation and recycling measures on the future Cr supply in the steel sector (Scenario S1–S5). Additionally, it explores the potential contributions of functional substitution and circular design strategies on future Cr demand and supply by incorporating two representative breakthrough technologies, namely additive manufacturing and hydrogen-based metallurgy, through a dedicated technology disruption scenario (S6). This scenario highlights additive manufacturing to reduce the total Cr demand by significantly enhancing material efficiency and enabling lightweight design [52], while hydrogen-based steelmaking, as a form of functional substitution, lowers the Cr alloying requirements for low- and medium-grade steels through improved baseline material properties [53]. The assumptions and parameters of these different scenarios are listed in Table 4.

**Table 4:** **Basic assumptions and parameter settings for various supply and demand scenarios**.

Scenarios	Basic assumptions and parameter settings
Baseline scenario (S1)	Resource use and recycling rates in the Cr industry will remain at 2020 levels without significant improvement (business as usual).
Resource conservation scenario (S2)	Resource utilization efficiency across all stages of the life cycle is expected to increase by 10% annually until reaching industry-leading standards. The target efficiencies are set as: 90% for chromite mining and refining [54], 93% for ferrochrome smelting [55], 95% for stainless-steel fabrication [56], and 95% for refractories and chemical products manufacturing [57].
High recycling growth scenario (S3)	The recycling rates for end-of-life products will increase 10% annually until they reach the international leading level of 95% [58].
Peak recycling efficiency scenario (S4)	The EoL recycling rate will rapidly increase to a peak level of 95% in 2024.
Combined policy scenario (S5)	This scenario integrates the advancements from both scenarios S2 and S4: Cr resource efficiency will increase by 10% annually, and the end-of-life recycling rate will increase to a peak level of 95 % since 2024.
Technology disruption scenario (S6)	The per capita Cr stock saturation levels will decline by 5% for infrastructure and mechanical equipment, 10% for electronic equipment and transportation, 3% for metal products and 12% for other uses across the baseline demand scenarios (LS, MS, HS) without additive manufacturing adoption [52,59]. Meanwhile, the material efficiency of ferrochrome and stainless-steel production will linearly increase to 98% by 2035 [60], and the Cr alloying ratio in other alloy steels (excluding stainless-steel and high-end alloys) will decrease linearly by 70% by 2035 [53]. Recycling assumptions in this scenario remain consistent with those in Scenario S3.

Under the low-saturation scenario (LS), the adoption of additive manufacturing will lead to a reduction in per capita Cr stock saturation levels to values (28.1 kg/person) below the 2023 baseline. Therefore, an inverse logistic function was employed, consistent with logistic-based approaches used in material stock dynamics [50].

In the steel sector, primary Cr sources can be replaced by secondary Cr resources. Such secondary resources can be continually sent back into the M&F (manufacturing and fabrication) stage through closed-loop recycling [58]. Eqs. (7)–(10) show how to calculate primary and secondary Cr supply.

(7) $$S_{t}\;=Q_{t}-R_{t}$$

(8) $$Q_{j,t}=\frac{F_{j,t}}{\prod_{i=1}^{3}Y_{i,j}}$$

(9) $$Q_{t}=\sum_{j=1}^{3}Q_{j,t}$$

(10) $$R_{t}=\frac{F_{out,t}}{r_{t}}$$

where $S_{t}$ represents the supply of primary Cr resources in year *t,* including both domestic production and imports; $Q_{t}$ represents the total demand for Cr resources in year *t*; $R_{t}$ represents the supply of secondary resources; $Q_{j,t}$ represents the demand for Cr resources in product type *j*, including stainless steel, other alloy steels, and refractories; $Y_{i,j}$ represents the resource utilization rate for Cr product type *j* at life cycle stage *i*, including chromite mining, ferrochrome smelting and F&M stages; $F_{out,t}$ represents the amount of Cr resources scrapped in year *t* and $r_{t}$ represents the EoL recycling rate of Cr products in year *t*.

### 2.3 Data sources

The historical data sources for Cr flows and stocks are consistent with our previous studies [9]. Specifically, the estimation of new scrap generated during the manufacturing stage is based on our field investigations at several leading recycling and stainless-steel production facilities in China. The obtained new scrap ratios were also cross-validated with values reported in the literature [58], and the differences were generally within an acceptable range (less than ±5%). For the stock-driven model, the stock saturation levels for different demand scenarios are based on several relevant studies [41,46,47] and governmental documents [61,62]. Population data are obtained from the China Statistical Yearbook [63], while future population forecasts are taken from the United Nations World Population Prospects 2022 [64]. Renewable energy capacities and material intensities are based on relevant reports and related studies [10,12]. Moreover, we interviewed some Cr experts working in several key Cr firms. They provided valuable and timely information and data and helped us fill some data gaps.

### 2.4 Uncertainty analysis

The uncertainties of this study are subject to statistical errors of input variables and the subjective nature of our assumptions. To assess the impacts of these uncertainties on MFA results, a Monte Carlo simulation was performed with 10,000 iterations to account such uncertainties by using a normal distribution [65]. Based on previous studies [19,66], we assume that uncertainties in statistical data (production, consumption and trade) are lower than those in model assumptions (Cr content and product lifespan). Thus, we set the standard deviations at 2% for statistical data and 5 % for model assumptions [67]. Results are listed in Fig. A1 of the Appendix.

## 3 Results

### 3.1 Overview of historical Cr flows and stocks

China’s Cr flows experienced a modest increase due to the stable growth of the stainless-steel industry during 2020–2023 (Fig. 4). The total imported Cr resource in the production stage increased by 20%, from 5102 Gg in 2020 (1655 Gg of FeCr and 3447 Gg of chromite ores) to 6145 Gg in 2023 (1853 Gg of FeCr and 4292 Gg of chromite ores). In the fabrication and manufacturing stage, > 84% of Cr was consumed by the stainless-steel industry in 2023, followed by other alloy steels (6%), refractory materials (4%), other metallurgical uses (4%) and chemical materials (2%). It is worth noting that the stainless-steel manufacturing process is characterized by the generation of substantial amounts of new scraps (e.g., production offcuts and machining remnants) and the recycled old scraps (e.g., discarded automotive parts and decommissioned construction materials from those EoL flows). Our estimates show that 986 Gg of Cr was recycled from old scraps in 2023, which can contribute to mitigating the demand for primary Cr resources.

**Fig. 4:**
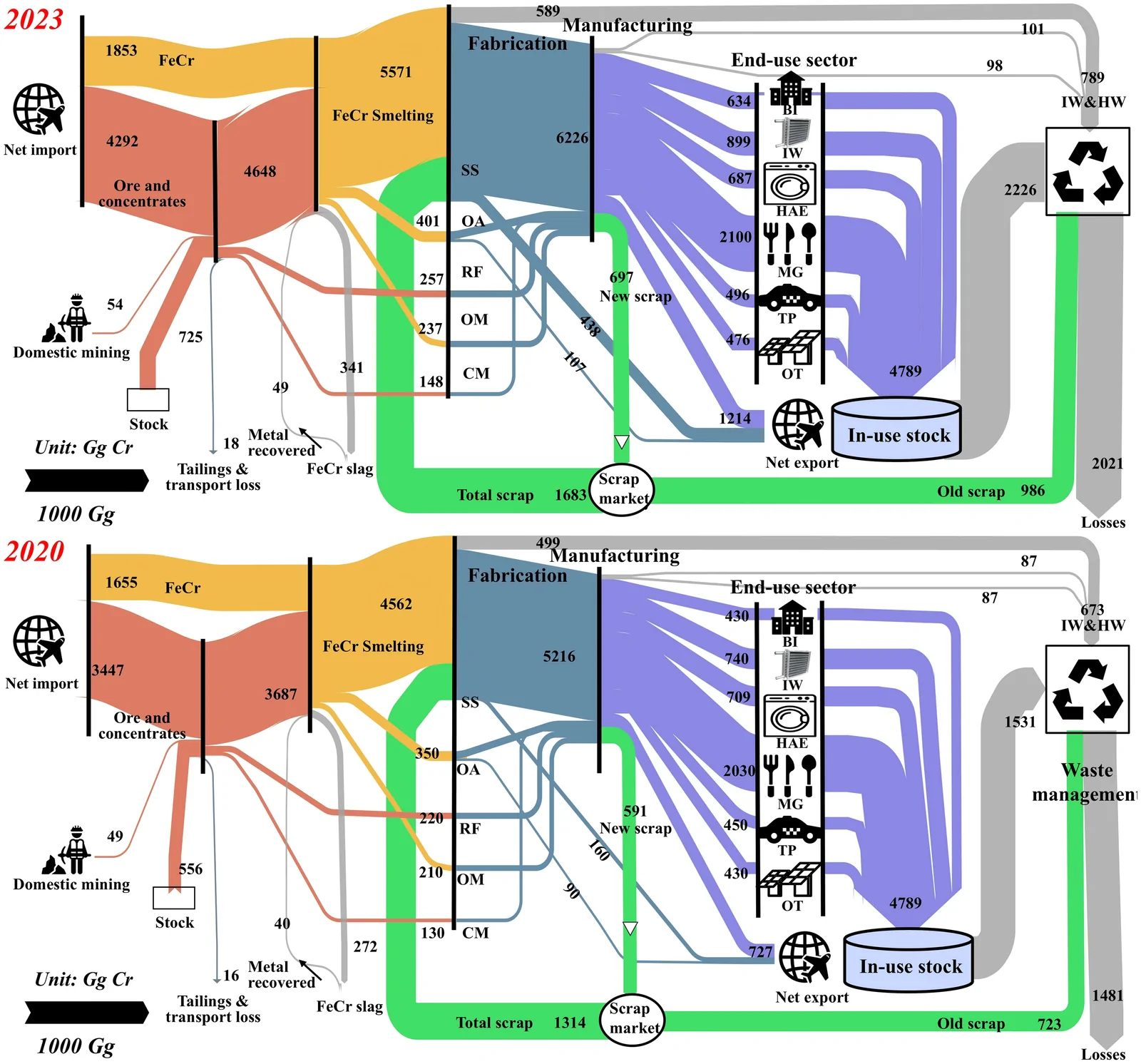
**Cr flows and stocks in China for the years 2020 and 2023**. The width of each flow is proportional to its corresponding value (Unit: Gg). SS, stainless-steel; OA, other alloy steel; RF, refractor materials; OM, other metallurgical uses; CM, chemical materials; IW, industry wastes; HW, hazardous wastes. BI, building and infrastructure; TP, transportation; HAE, household appliance and electronics; IM, industrial machinery; MG, metal goods; OT, others.

The structure of Cr flows has remained relatively stable in China throughout this study period. But a persistent and significant mismatch exists between the domestic mineral supply and the production capacity of downstream products, such as ferrochrome and stainless steel, which results in more concerns about Cr supply security. Approximately 50 Gg of Cr was annually supplied from domestic chromite, accounting for <1% of the imported Cr resources. The total domestic Cr supply (both primary and secondary Cr) accounted for only 14% of the national total Cr consumption in 2023. International trade also played a critical role on the export side. In 2023, the net export of Cr embedded in semi-finished and finished products reached 1214 Gg, representing a 67% increase from 2020 and indicating a strong post-pandemic economic recovery. This imbalance suggests that China’s Cr supply could greatly disrupt the global Cr supply chain and affect those countries with rich Cr resources.

China consumed 5291 Gg of Cr in various sectors in 2023, making it the world’s largest Cr consumer. The largest end-use category is metal products (42%), followed by industrial machinery (17%), household appliances and electronics (15%), building and infrastructure (12%), others (10%), and transportation (9%). Additionally, the EoL flows increased dramatically from 1531 Gg in 2020 to 2226 Gg in 2023, representing an increase of 45%. Such growth was primarily attributed to the retirement of stainless-steel products. Many Cr-containing products reached the ends of their service years during this period (typically 10–15 years) [14].

### 3.2 Projections of future Cr in-use stocks, inflows and EoL flows

Fig. 5 illustrates the historical trends of Cr in-use stocks in the steel industry from 2000 to 2023, along with projected trajectories under different scenarios for the period of 2024–2050. The in-use stocks initially experienced rapid growth (acceleration phase), followed by a deceleration (stabilization phase), and then gradually declined after reaching the peak (decline phase). The in-use stocks experienced rapid growth before 2013, with an annual growth rate of > 16%. This trend echoed China’s rapid industrialization and urbanization, which induced higher industrial output and infrastructure expansion. In addition, the increasing demand for household appliances, such as air conditioners and refrigerators, further accelerated the accumulation of Cr within the socio-economic system [68]. However, the growth rate of Cr in-use stock began to decelerate since China entered its economic New Normal in 2013, marked by a shift from high-speed growth to more sustainable, moderate growth [69], reaching approximately 8% in 2023. The forecast results indicate that the growth rate is expected to further decrease over the next 4–6 years until the in-use stock peaks.

**Fig. 5:**
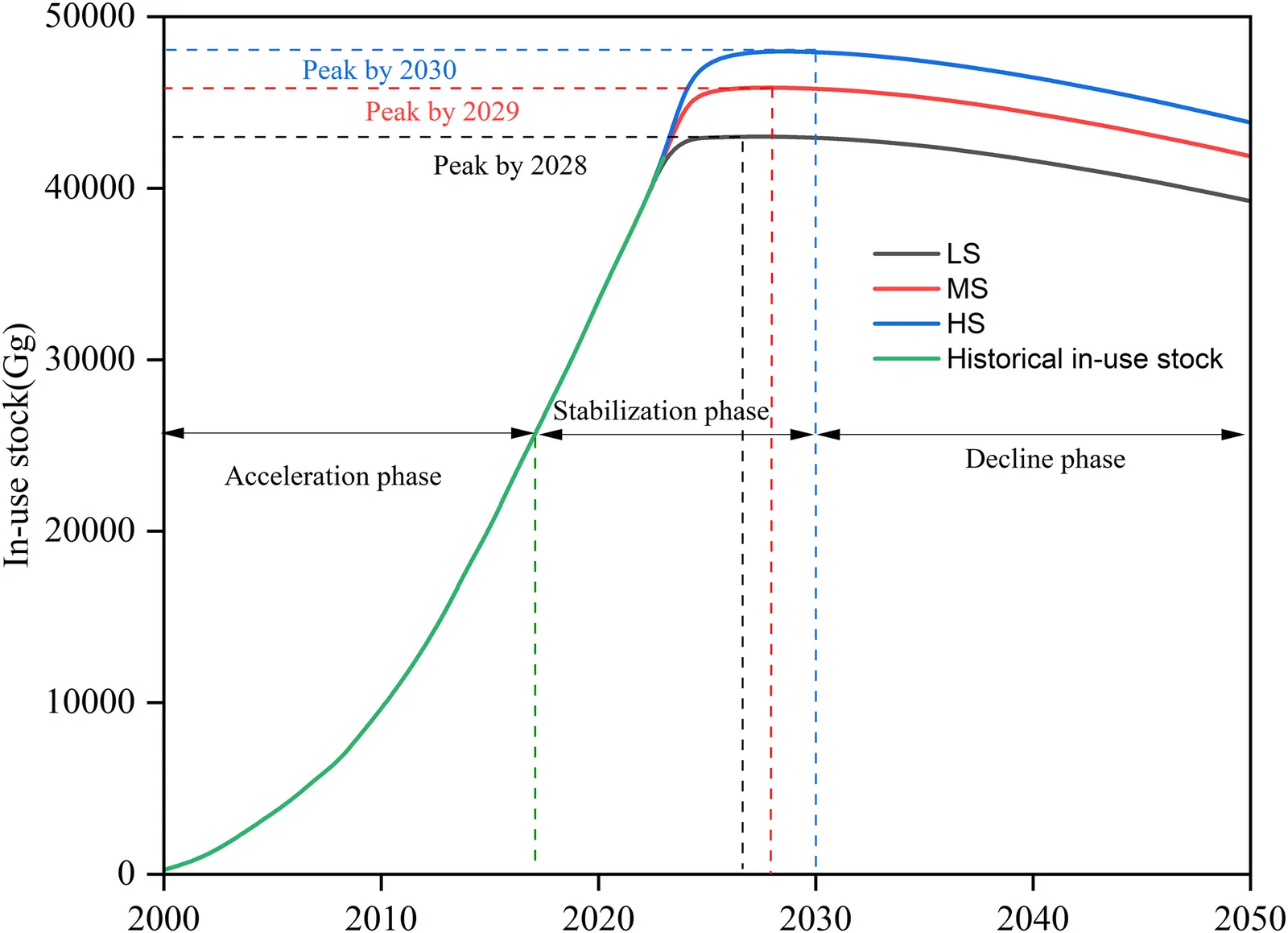
**Trends in Cr in-use stock in the steel industry across different scenarios (2000–2050)**.

Our projection results indicate various peak levels of in-use stock under three scenarios: low-saturation (LS), medium-saturation (MS), and high-saturation scenarios (HS). The in-use stock is projected to peak at 45,872 Gg in 2027, 45,865 Gg in 2028, and 47,974 Gg in 2029 under these three scenarios, respectively. The anticipated population decline is the primary reason for the reduction of Cr stock [70]. When the in-use stock peaks, the annual EoL flow will exceed the annual inflow during the in-use stage, implying that the total secondary Cr resource from EoL products will surpass the total demand for Cr. This means that circular economy measures should be taken to make sure that adequate secondary Cr resources can be collected [6].

Fig. 6 illustrates the trends of Cr inflows (demands) and outflows (EoL flows) during the in-use phase under three scenarios from 2024 to 2050. It is evident that the demand for Cr in all sectors will decline sharply during 2024–2027 as both per capita in-use stock and population reach their peak levels. After the peak of in-use stock, Cr inflows could be gradually replaced by the recycled Cr from those EoL products. Specifically, the inflow for the metal goods sector will fluctuate after 2026, which can be explained by their average lifespans of 10 years. Those metal products made in 2016–2024 will reach the ends of their lifespans, meaning that the demand for Cr in this niche market will increase during 2026–2034.

**Fig. 6:**
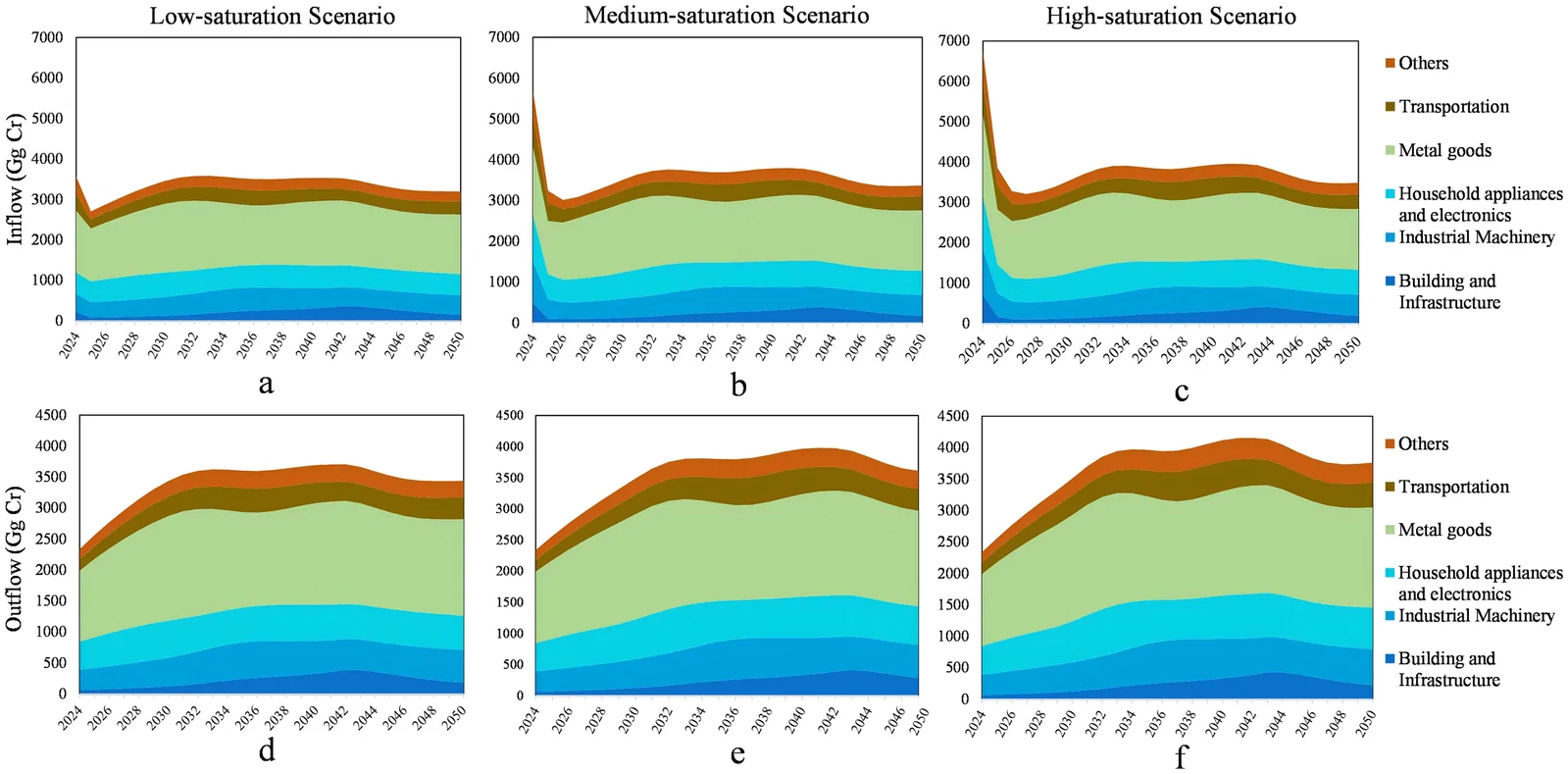
**Trends of Cr inflows (demands) and outflows (EoL flows) of the in-use stage under three different scenarios (2024–2050):** (a) inflow under LS, (b) inflow under MS, (c) inflow under HS, (d) outflow under LS, (e) outflow under MS, (f) outflow under HS.

Meanwhile, the total EoL flow will increase from 2024 to 2034, indicating great pressure to effectively collect these EoL products. Such EoL flow will be twice the size of the 2021 level, reaching 3592 Gg under the LS scenario and 3947 Gg under the HS scenario, respectively. The total EoL flow will peak in 2043, ranging from 3574 Gg (under the LS scenario) to 4188 Gg (under the HS scenario). Such EoL flow will gradually decline until 2050 and remain stable, ranging from 3060 Gg (under the LS scenario) to 3427 Gg (under the HS scenario) in 2050.

Fig. 7 illustrates the projected demand for Cr in various renewable energy fields from 2021 to 2050. The results indicate that the highest demand for Cr will take place in wind power, except for the period of 2036–2040. From 2021 to 2035, the Cr demand for Cr in wind power is projected to increase steadily. Another interesting finding is that the demand for Cr in geothermal power will greatly increase during 2036–2040, with an annual demand of 97 Gg. This jump is mainly due to increasing geothermal power installation and its higher material intensity. The geothermal development is expected to surge during 2036–2040 [50]. However, after 2040, the growth of geothermal installations is projected to stabilize due to resource constraints and increased competition from more cost-competitive renewable sources such as wind and solar power [71]. The total annual demand for Cr in these renewable energy sources will peak at 136 Gg during 2036–2040, but accounting for only 1/20 to 1/30 of the demand for Cr in the steel sector during the same period.

**Fig. 7:**
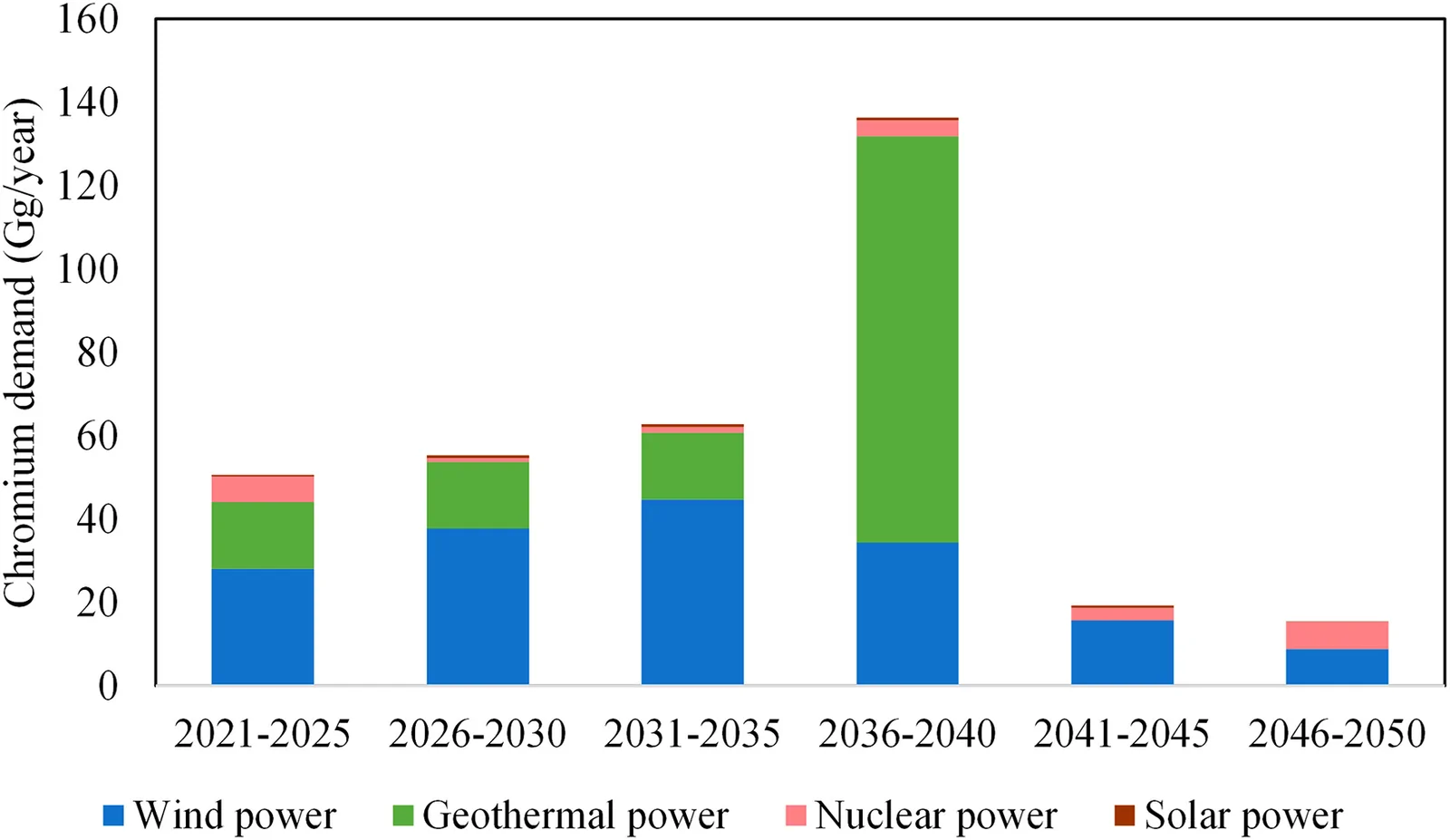
**Cr demand in the renewable energy sector from 2021 to 2050**.

Fig. 8 shows the total demand for Cr from 2024 to 2050, which combines the demands for Cr in the steel, chemical, other metallurgical, and renewable energy sectors. The results show that the total Cr demand under all three scenarios mirrors the trend observed in the steel sector since the combined demand for Cr from chemical, other metallurgical, and renewable energy sectors is significantly lower than that of the steel sector. Due to the reduced per capita stock growth in this steel sector and the reduced total population growth, the demand for Cr will experience the most volatile period during 2024–2026. Both per capita stock and the total population will peak in 2026, implying that the demand for Cr will mainly be driven by the replacement of obsolete products, and such demand will fluctuate until 2050.

**Figs. 8:**
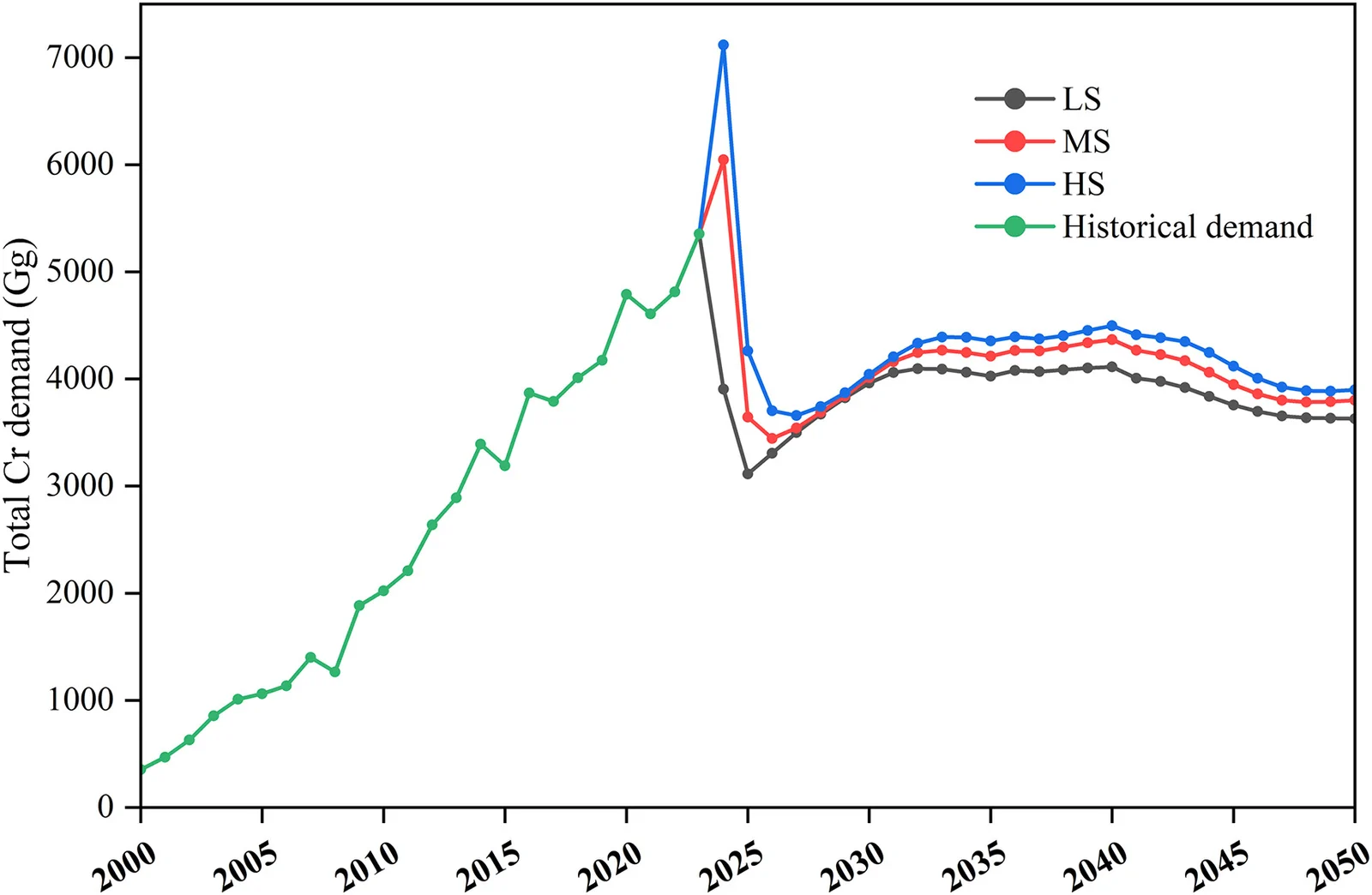
**Total demand for Cr under different scenarios from 2000 to 2050**.

### 3.3 Future Cr supply and demand pathways under different scenarios

Fig. 8 shows the supply pathways of both primary and secondary Cr resources in the steel sector under different scenarios, reflecting the effects of different circular economy strategies. Under the Baseline scenario (S1), resource use and recycling rates of Cr will remain at 2020 levels, which is a business-as-usual reference. In contrast, the Resource conservation scenario (S2) assumes improved resource efficiency throughout the life cycle. Under this scenario, China’s total demand for Cr is expected to decline due to reduced losses during various life cycle stages. As a result, the cumulative demand for primary Cr resources would decrease by 11% between 2021 and 2050. However, this relatively limited reduction underscores that conservation alone will be insufficient to reduce China’s heavy reliance on primary resources.

Under a high recycling growth scenario (S3), which assumes a 10% annual increase in the EoL recycling rate until reaching 95%, the supply of secondary Cr resources would significantly increase during 2024–2032, leading to a gradual decoupling between primary Cr resources supply and the total demand for Cr. The supply of secondary Cr resources will surpass the supply of primary resources for the first time in 2028 and then stabilize at 3188–3489 Gg by 2050. Meanwhile, the supply of primary Cr resources would decline to 720–780 Gg by 2050, representing approximately 11% of China’s total primary Cr consumption in 2023. The peak recycling efficiency scenario (S4) accelerates this transition. With the EoL recycling rate reaching 95% by 2024, secondary Cr overtakes primary supply as early as 2026. Compared to S3, China’s reliance on primary Cr resources may be sharply reduced to 26−33% within a short period, demonstrating a significant short-term impact of rapid recycling improvements. These results highlight the critical role of recycling in reducing dependence on primary resources and enhancing the sustainability of China’s Cr supply.

The combined policy scenario (S5) recognizes the significant effects of integrating the efforts of improving resource efficiency (as in S2) and EoL recycling on the future Cr supply (as in S4). Secondary Cr resources will play a dominant role in meeting the domestic demand after 2026 under this scenario. The consumption of primary Cr resources will further decline compared to scenarios S2 and S3, stabilizing at 315–337 Gg by 2050. Assuming domestic chromite supply remains at 2023 levels, China’s import reliance^2^ on Cr resources is expected to decrease to 8% by 2050. This scenario reflects the most ambitious outcome of supply-side circular economy strategies in reducing dependence on imported Cr.

The technology disruption scenario (S6) represents a combined strategy integrating demand-side interventions with enhanced recycling performance and resource utilization rate. Under this scenario, the widespread adoption of additive manufacturing and hydrogen-based steelmaking will further decrease the primary Cr supply in all saturation pathways compared to the improvements achieved through enhanced recycling alone (S3). This reduction is largely driven by lightweight product design enabled by additive manufacturing, which lowers the overall demand for Cr-intensive inputs. As a result, primary Cr supply would decrease to merely 129–158 Gg by 2050, which is approximately one-fifth of the 2023 level. At that point, the dependence on imported resources would drop to as low as 3%, indicating the formation of a nearly self-sufficient domestic resource loop. Among all scenarios, S6 achieves the most balanced and effective transition toward circularity.

## 4 Discussion

### 4.1 Uncertainties and limitations

Results indicate that the uncertainties of statistical data and model assumptions are acceptable, which would not significantly affect the key findings of this study (Fig. A1). The uncertainties of recycled old scraps are relatively higher, ranging from −13.5% to 12.1%. In this study, the recycling flows are estimated by measuring the gap between the primary input of FeCr and the output of stainless steel. Based on the principle of mass conservation, this assumption is influenced by technical parameters related to production, trade, stock and consumption. The uncertainties of recycling flows could be reduced in the future when more comprehensive statistical data on these flows are available.

The scenario analysis results in this study further help measure the impacts of potential variables on Cr metabolism. By referring to the current patterns of resource utilization in developed countries and the emerging demand for Cr from renewable energy, this study addresses the main uncertainties in the future Cr supply and demand under multiple scenarios. Although these scenarios can facilitate the estimation of different pathways, it will be necessary to consider other scenarios that incorporate other emerging technologies (such as blockchain technology for supply chain transparency and resource management) so that more Cr supply and demand dynamics can be projected. In addition, due to the lack of some relevant data, we had to use some data collected from our site visits, which are based on one individual firm and the estimation of relevant experts. We expect that future studies may collect more reliable and accurate data for their analysis.

In addition to technological and sectoral demand dynamics, the global Cr supply chain is vulnerable to geopolitical and market uncertainties, particularly given the concentration of Cr mining production in countries such as South Africa and Kazakhstan [14]. Export restrictions or political instability in these regions may result in significant supply disruptions and price volatility. While such factors are not modeled in this study, our scenario-based approach offers practical pathways to reduce dependence on external sources and enhance supply security. Future research could further enrich this analysis by incorporating a market equilibrium framework to simulate price feedback effects and assess the implications of potential trade policy shocks [72].

### 4.2 Strengthening governance on chromium demand

According to our results, the per capita in-use Cr stock significantly increased from 0.2 Gg in 2000 to 29.3 Gg in 2023, which is a 140-fold rise (Fig. A2). Such a stock peaked in 2023, which is higher than the peak value of 26 Gg in the United States in 1992, which serves as the reference point for the low saturation (LS) scenario. The rapid growth in Cr demand prior to 2023 is further supported by recent findings from Yang et al. [30], who conducted a product-level substance flow analysis of chromium in China’s stainless-steel supply chain between 2012 and 2022. Actually, after the demand for Cr saturates, such a stock will continue to rise and peak at 30 Gg in 2050. This long-term growth trajectory and saturation pattern are consistent with previous studies [44,45], which forecasted sustained growth in Cr demand before entering a stable phase. A sharp Cr demand decline may result in Cr oversupply, leading to price volatility and reduced incentives for Cr mining and refining activities. This pattern has been observed for other critical resources, such as lithium, where rapid demand fluctuations triggered significant price swings and supply chain disruptions [67]. In response to such an issue, policymakers should prepare appropriate measures to balance Cr supply and demand, such as adjusting production capacities or initiating strategic reserves that can adapt to dynamic demand.

The demand for Cr began to decrease in both the United States and Japan after their per capita GDP reached $25,000 [44]. China will not reach this threshold until 2025, the year in which Cr demand is projected to approach its peak, given that its GDP per capita was around $12,000 in 2023. In developed countries, such as the United States, Japan, Germany, France, and the United Kingdom, the shift to service-based economies has resulted in their lower Cr demand [45,73]. In contrast, the scales of China’s stainless-steel and ferrochromium industries contribute significantly to the global production, leading to its higher demand for Cr [14,68]. These structural differences underscore a need for differentiated national strategies in resource governance. Yet, such heterogeneity is often overlooked in the global assessments [74].

This disparity highlights an urgent need for China to adopt targeted demand-side strategies to manage its growing Cr demand. Beyond conservation and recycling, other circular economy approaches, such as reduction and substitution, should be supported to reshape the national Cr demand structure. Although Cr remains irreplaceable in core sectors like stainless steel and alloy steels under current technological conditions, policy support for advancing emerging technologies and facilitating substitution could contribute to demand reduction. As indicated by technology disruption scenario (S6), the lightweight product design enabled by the widespread adoption of additive manufacturing would mitigate the overall demand in all end-use sectors, leading to less reliance on primary Cr supply (Fig. 9). In comparison, the gains from improved resource efficiency through additive manufacturing and the substitution potential of hydrogen-based steelmaking are smaller. Nevertheless, they offer supplementary benefits by reducing material losses and lowering the Cr alloying intensity required in low- and medium-grade steels. Together, these upstream interventions demonstrate the potential of reduction and substitution strategies to decouple Cr demand from economic growth. This finding reinforces the argument made by Liang et al. [74], who emphasized the importance of integrating technological advancements and evolving material intensities into scenario analysis, an aspect that is often overlooked in traditional demand projections. However, the realization of such a transition depends on the timely deployment of enabling technologies by 2035. To that end, coordinated industrial policies, sustained investment in research and development, and systemic upgrades to production systems and supply chains are essential to accelerate the implementation of circular economy strategies.

**Fig. 9:**
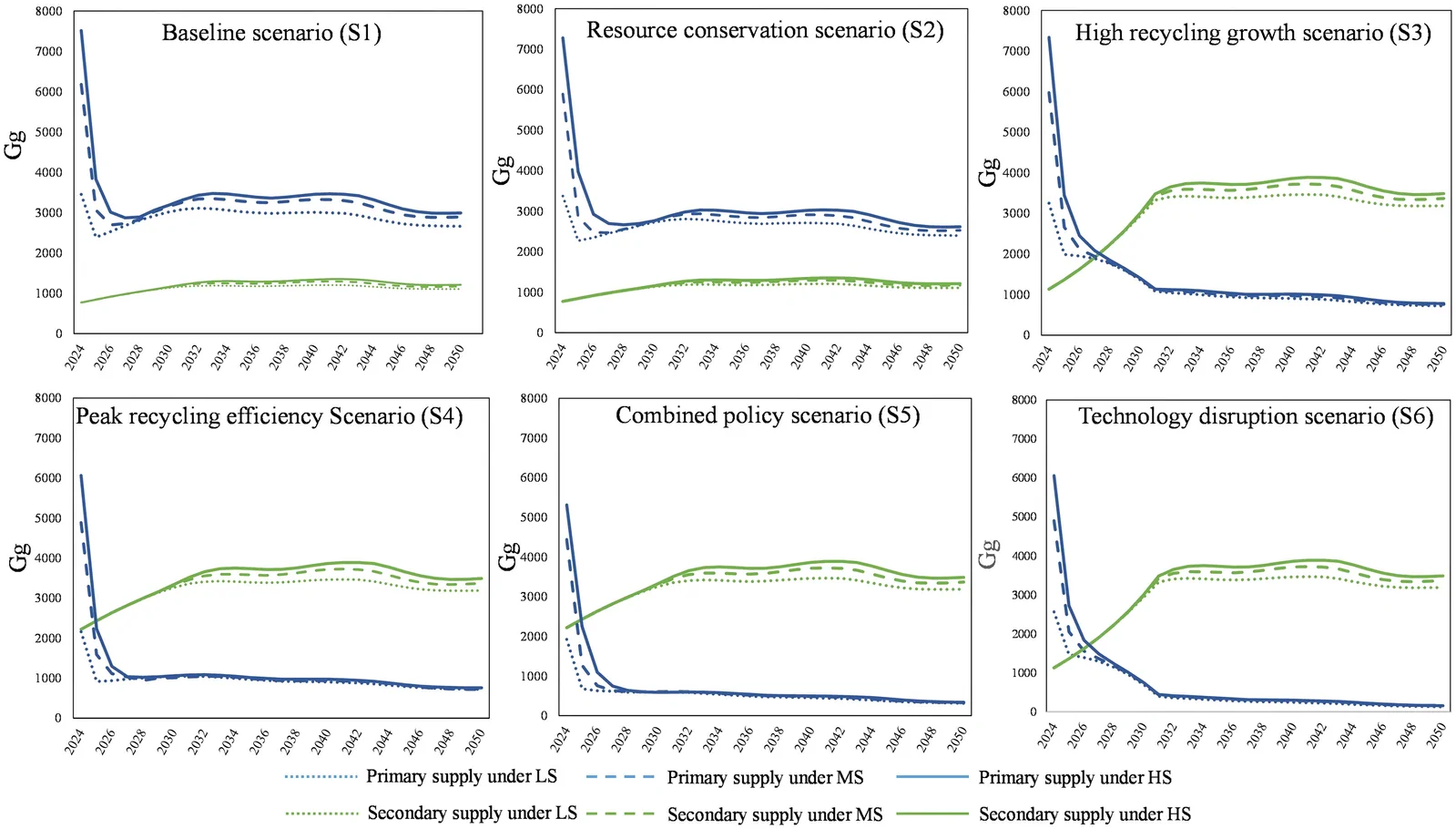
**Supply pathways of primary and secondary Cr resources under different scenarios (2024–2050)**.

Furthermore, although the absolute demand for Cr from renewable energy remains small compared to that of steel sector, the temporal concentration of demand in high-intensity technologies such as geothermal still warrants attention. This is particularly relevant when planning long-term decarbonization strategies that may involve rapid deployment of multiple renewable energy technologies simultaneously. As noted by relevant studies [75,76], small individual demands can accumulate into significant resource pressures when upscaled globally. Therefore, even for metals that are not typically classified as “critical” in the energy transition context such as Cr, it is still important to include them in scenario-based material assessments to avoid overlooking niche but time-sensitive demand surges.

### 4.3 Improving chromium recycling

It is essential to improve chromium recycling along the entire Cr supply chain so that the sustainable supply of Cr can be ensured. In this study, an optimistic EoL recycling rate of 95% is assumed under the peak recycling efficiency scenario (S4), and set as the final target for both the high recycling growth scenario (S3) and the combined policy scenario (S5). This target can only be achieved when those discarded stainless-steel products are fully recycled and mature recycling technologies are sufficiently applied. However, it is challenging to achieve such an ideal rate in a short time. Our results indicate that the EoL recycling rate has been only slightly improved from 33% in 2020 to 34% in 2023, significantly lower than those in developed countries [14,58].

One barrier that restricted the EoL recycling is the prevalence of informal recycling in China. Our results shows that over 60% of the EoL flows were originated from retired metal products and electronic appliances, many of which came from households. Currently, most of such EoL products are collected by unregulated entities, such as informal scavengers and small recycling companies. These entities do not have mature and effective recycling technologies and are not under the governmental governance [77]. The potential increase of EoL flow, particularly between 2024 and 2034, may lead to more available secondary Cr resources. Although the Chinese government reduced the tax burden on formal recycling enterprises in 2021 [78], more supportive policies should be released to fully support the growth of such formal recycling sector so that the higher recycling rates under scenarios S3 and S5 can be achieved, such as the strict enforcement of relevant regulations, the phaseout of recycling firms without official licenses, the national Cr recycling standards, and necessary capacity-building efforts.

Another barrier that restricts Cr recycling is the relatively low share of Electric Arc Furnace (EAF) in the steelmaking industry. Such furnaces can deal with secondary Cr resources in the stainless-steel sector, but there are still few industrial applications. Although this share is expected to rise to 30% by 2030 [79], it is still impossible to meet the high recycling demand under scenario S3, where secondary Cr resources may play a dominant role in Cr supply. Consequently, it is urgent to adjust this share by encouraging more investment in this specific field and more research & development activities to further improve the overall Cr recycling efficiency. Importantly, our scenario analysis also suggests that the effectiveness of EAF-based recycling can be substantially enhanced when coupled with breakthrough technologies (e.g., hydrogen-based steelmaking) modeled in the technology disruption scenario (S6). As a low-carbon alternative to conventional blast furnace processes, hydrogen metallurgy not only reduces greenhouse gas emissions but also enables functional substitution by enhancing baseline material performance, thereby lowering chromium alloying requirements [53].

The significance of recycling also has broader relevance beyond China. As highlighted by Baldassarre [80], circular economy approaches are increasingly recognized not only for their environmental benefits but also as strategic tools to improve material security under the contexts of the global supply chain disruptions. For instance, many European countries having reached their demand saturation and now possess substantial EoL flows. However, these countries are facing declining domestic extraction and rely on importing Cr resource [81]. Our scenario analysis results offer valuable insights to such Cr resource-dependent economies so that they can strengthen their supply resilience through domestic recycling.

## 5 Conclusions

As one of the critical minerals, chromium (Cr) is widely used in metallurgical, chemical and renewable energy sectors in China. However, China currently relies on importing such resources and has to face a supply risk. It is essential for China to consider both primary and secondary Cr resources to enhance its supply, which should be based upon a full understanding on the current status of Cr resource efficiency and its future supply-demand dynamics. This study fills this research gap by examining the Cr metabolism features along its supply chain in China for the period of 2020–2023. Also, future Cr supply and demand pathways are projected for the period of 2024–2050 through scenario analysis. Results indicate that the total Cr flows and stocks increased slightly from 2020 to 2023. Domestic Cr supply (including both primary and secondary Cr supply) accounted for 14% of the total Cr consumption in 2023, while the EoL flow increased dramatically from 1531 Gg in 2020 to 2226 Gg in 2023. Unfortunately, few secondary Cr resources were recovered from these EoL products.

A major finding in this study is that traditional sectors, such as stainless steel and other alloy steels, will influence the future Cr demand and supply. The demand for Cr in the steel sector will peak in 2024 since both per capita in-use stock and the total population peak in the same year. Such demand will remain stable between 3060 Gg and 3427 Gg during 2024–2050. This demand reduction may disrupt the future Cr supply chain. It is therefore critical to establish a robust monitoring and early warning system so that the associated supply risk can be mitigated.

Another significant finding is that the total EoL flow is expected to continuously increase from 2024 to 2034 and surpass the inflow of in-use stage between 2028 and 2030. This secondary Cr supply and demand dynamics has been neglected in previous studies and implies a potential supply security. Scenario analysis results further show that China’s reliance on importing Cr would decrease to 8% by 2050 if its EoL recycling rate can reach the global leading level of 95% and the primary Cr resource efficiency can be improved across various stages of the entire Cr industrial chain. The demand reduction and substitution strategies could further reduce China’s net import dependence to just 3% by 2050. These findings highlight an urgent need for an integrated circular economy approach that combines improved recycling, enhanced resource efficiency, and demand-side reduction strategies to ensure the long-term security of China’s Cr supply. These insights provide valuable policy implications to all the resource-dependent economies so that they can prepare their resource management policies to ensure their Cr supply.

## CRediT authorship contribution statement

**Ziyan Gao:** Writing – original draft, Visualization, Validation, Software, Methodology, Investigation, Formal analysis, Data curation, Conceptualization. **Yong Geng:** Writing – review & editing, Writing – original draft, Validation, Supervision, Resources, Project administration, Methodology, Funding acquisition, Formal analysis, Data curation, Conceptualization. **Wenqiu Cai:** Writing – review & editing, Writing – original draft, Formal analysis, Data curation.

## Declaration of competing interest

The authors declare that they have no conflicts of interest in this work.

## Appendix Supplementary materials

Image, application 1

## Footnotes

1 The chemical sector and other metallurgical uses consume only 2−3% of the total chromium resources, with dispersed applications. Their demand is projected using a linear regression model extrapolated to 2030, after which it is assumed to remain at that level.

2 Import reliance is defined as the proportion of imported chromium resources relative to the total chromium supply.
