# Supplementary material for: Securing China’s chromium supply through circular economy
Source: Fundam Res. 2025 Nov 20;6(5):3340–51. doi: 10.1016/j.fmre.2025.11.010 (PMC13644475; doi:10.1016/j.fmre.2025.11.010)
Supplement: Supplementary file 1 [file mmc1.docx]

# Appendix

## 1. The calculation of outflows

The outflows during the in-use stage were quantified by assigning a lifetime distribution function to specific end-use sectors ^[1]^. Weibull probability distribution model was applied to estimate Cr outflows its superior adaptability to various types of lifetime data ^[2,3,4]^. The probability that a product discarded after *t* years of use is given by the following equation: $\begin{aligned} p^{t}=\exp\left( -\left( \frac{t-a}{\beta} \right)^{\alpha} \right)-\exp\left( -\left( \frac{t-a+1}{\beta} \right)^{\alpha} \right), a\leq t\leq b\#\left( A1 \right) \end{aligned}$

where $p^{t}$ represents the probability that products of a certain category have a lifetime of 𝑡 years; *a* and *b* are the minimum and maximum lifetimes of Cr-containing products, respectively; $\alpha$ and $\beta$ are the scale and shape parameter that characterize the Weibull distribution.

To determine $\alpha$ and $\beta$, we assumed that 99.7% of all Cr-contained products reach the end of their lifespan within the range [a,b]. Thus, the corresponding cumulative distribution function can be expressed as:

$\begin{aligned} \mathrm{IP}\left( a\leq t\leq b \right)=1-\exp\left( -\left( \frac{t-a}{\beta} \right) \right)^{\alpha}=\gamma\#\left( A2 \right) \end{aligned}$

where $\gamma=99.7\%$, *a* and *b* are pre-specified.

In addition to Eq. A2, the mode *m* of the Weibull distribution is also utilized to determine the values of $\alpha$ and $\beta$. The mode m corresponds to the point at which probability density function reaches its maximum, representing the most likely observed value: $\begin{aligned} m=a+\beta\left( \frac{\alpha-1}{\alpha} \right)^{\frac{1}{\alpha}}, \alpha\geq1\#\left( A3 \right) \end{aligned}$

Given the values of *a*,*b* and *m*, the parameter $\alpha$ and $\beta$ can be calculated by solving Eq.A2,A3. Subsequently, $f_{t,out}$ in Eq.2 of the main text can be determined.

The data used for modeling Weibull distribution, including both lifetime intervals [a,b] and modal value *m*, are set according to previous studies ^[4,5,6]^. **Table A1** presented the data used and parameters calculated for modelling lifetime distribution of each category.

**Table A1**. Lifetime distribution of various chromium end-use categories in China.

| Period | Sector | Lifetime intervals | | Most likely lifetime | Weibull parameters | | | Data sources |
| --- | --- | --- | --- | --- | --- | --- | --- | --- |
|  |  | *a* | *b* | mode *m* | *α* | *β* | |  |
| 1960-1990 | Household appliances and electronics | — | — | — | — | | — |  |
|  | Industrial machinery | 10 | 30 | 20 | 3.1 | | 11.34 | (Zhang et al., 2015)^[6]^ |
|  | Transportation | 10 | 25 | 19 | 4.01 | | 9.67 | (Zhang et al., 2015) ^[6]^ |
|  | Building and infrastructure | 23 | 40 | 30 | 2.54 | | 8.52 | (Zhang et al., 2015) ^[6]^ |
|  | Metal goods | — | — | — | — | | — |  |
|  | Others | 1 | 35 | 15 | 2.7 | | 16.9 | (Daigo et al., 2010) ^[5]^ |
| 1990-present | Household appliances and electronics | 5 | 15 | 9.3^a^ | 2.65 | | 5.14 | (Wen and Ji, 2013) ^[4]^ |
|  | Industrial machinery | 10 | 30 | 15 | 1.84 | | 7.67 | (Zhang et al., 2015) ^[6]^ |
|  | Transportation | 10 | 25 | 15 | 2.17 | | 6.66 | (Zhang et al., 2015) ^[6]^ |
|  | Building and infrastructure | 19 | 40 | 21 | 1.34 | | 5.62 | (Zhang et al., 2015) ^[6]^ |
|  | Metal goods | 5 | 15 | 9.8 | 2.83 | | 5.37 | (Gao et al, 2021) ^[7]^ |
|  | Others | 1 | 35 | 14.24 | 2.7 | | 16.9 | (Daigo et al., 2010) ^[5]^ |

## 2. The saturation level of per capita in-use stock

In the low-demand scenario, the per capita stock saturation level is set at 30 kg/cap. This value is derived from the “S” curve of chromium consumption observed in the United States, which demonstrates a relatively lower trend. Similar consumption patterns have been documented in other developed countries, such as Germany, France, and the United Kingdom ^[8]^. Historically, the U.S. produced significant quantities of chromium ore from 1900 to 1962, with notable peaks during World War I and World War II, placing it among the leading global producers. From 1974 to 1997, the U.S. continued produce ferrochrome, but production ceased thereafter, rendering the country almost entirely reliant on imports for chromium products. In the early 1990s, U.S. demand for stainless steel exhibited characteristics of stagflation. Compared to Japan, the per capita stainless steel stock saturation level in the U.S. remains lower, estimated at approximately 22 kg/cap ^[9]^. This value is slightly below the per capita stock of chromium resources in use in China in 2020, which stood at 24 kg/cap. To account for historical fluctuations and ensure data robustness, this study sets the stock saturation level at 26 kg/cap for the low-demand scenario, positioning it marginally above China’s current level to reflect a conservative, lower saturation condition.

In the medium-demand scenario, the per capita stock saturation level is primarily informed by Japan's experience, taking into account both economic development and industrial structure. The reasons are three folds. First, there are numerous parallels between China and Japan regarding their economic development trajectories ^[10]^. Both countries have adopted government-led economic systems, achieving industrial advancements and strategic global integration through the formulation of medium- and long-term plans. Furthermore, both nations have emphasized the cultivation of scientific talent and workforce development, initially capitalizing on inexpensive labor as a key competitive advantage. Second, in terms of stainless-steel industry development, China and Japan also exhibit several similarities. Both countries initiated stainless steel production in the early 1950s and have since become major global producers. Although Japan produced chromium ore from 1910 to 1993, its output remained relatively limited. Additionally, Japan produced ferrochrome from 1974 to 2020, with production declining from 542 kilotons in 1974 to just 13 kilotons in 2020. This decline reflects Japan's transition into a phase of stagnant growth in stainless steel demand, alongside fluctuations in chromium production over recent decades. Based upon the factors of economic development, industrial similarities and chromium production trends observed in Japan, this study sets the per capita stock saturation level for the medium-demand scenario at 32 kg/cap.

In the high-demand scenario, the stock saturation level is set at 33.5 kg/cap, primarily based on the experience of South Korea, which follows a high S-shaped curve in chromium consumption ^[8]^. Around 2015, chromium consumption in South Korea peaked and subsequently began to decline along this curve. Previous studies have indicated that, before reaching its peak, South Korea's per capita stainless-steel stock was approximately 1.5 times that of the United States during the same period ^[11]^. Consequently, the per capita saturation level in the high-demand scenario is set at 33.5 kg, equivalent to 1.5 times the U.S. saturation level of 22 kg. It is worth noting that other countries with high S-shaped curves, such as Sweden and Finland, have even higher per capita stainless-steel stocks than South Korea. However, due to their long histories of stainless-steel production and relatively small populations, these countries were not considered when setting the high-demand scenario.

Moreover, this study assumes that the share of each sector in total stock per capita will remain largely consistent with the 2023 levels across high, medium, and low demand scenarios. Despite these consistent shares, the overall volume is expected to rise in next 5 years. This trend aligns with the sector planning in China’s stainless-steel consumption and the overall economic development strategy.

## 3. Uncertainty analysis results

Figure A1 illustrates the uncertainties of main results of MFA. The solid lines show the original results. The error bars represent the uncertainty range. Figure A1 Uncertainty analysis results of historical chromium flows and stocks in China: (a) Total net imports of Cr along the life cycle; (b) Inflows of in-use stock; (c) Recycled old scraps; (d) EoL flows.

## 4. Per capita in-use stock

Figure A2 The trend of per capita in-use Cr stock during 2000-2023.^[1]^

## Reference

[1] Melo. M. T, Statistical analysis of metal scrap generation: the case of aluminum in Germany. Resources, Conservation and Recycling, 1999, 26(2):91–113. <https://doi.org/10.1016/S0921-3449(98)00077-9>.

[2] Wang. M, Liang. Y, Yuan. M, et al. Dynamic analysis of copper consumption, in-use stocks and scrap generation in different sectors in the U.S. 1900–2016. Resources, Conservation and Recycling, 2018, 139:140–149. <https://doi.org/10.1016/j.resconrec.2018.07.022>.

[3] Wang. Q-C, Wang. P, Qiu. Y, et al. Byproduct surplus: lighting the depreciative europium in China’s rare earth boom. Environmental Science & Technology, 2020, 54(22):14686–14693. <https://doi.org/10.1021/acs.est.0c02870>.

[4] Wen. Z, Ji. X, Copper resource trends and use reduction measures in China. Journal of Tsinghua University (Science and Technology), 2013, 53, 1283–1286. <https://doi.org/10.16511/j.cnki.qhdxxb.2013.09.005>.

[5] Daigo. I, Matsuno. Y, Adachi. Y. Substance flow analysis of chromium and nickel in the material flow of stainless steel in Japan. Resources, Conservation and Recycling, 2010, 54(11):851–863. <https://doi.org/10.1016/j.resconrec.2010.01.004>.

[6] Zhang. L, Yang. J, Cai. Z, et al. Understanding the Spatial and Temporal Patterns of Copper In-Use Stocks in China. Environmental Science & Technology, 2015, 49(11):6430-6437. <https://pubs.acs.org/doi/10.1021/acs.est.5b00917>.

[7] Gao. Z, Geng. Y, Zeng. X, et al, Evolution of the anthropogenic chromium cycle in China. Journal of Industrial Ecology, 2021. https://doi.org/10.1111/jiec.13207.

[8] Zhang. Z, Pan. Z, Che. D, Yan. Q, Wu. Q, 2023. Chromite ore resources characteristic and analysis of supply and demand situation from 2021 to 2035 in China. Geology in China, 51(4): 1191−1209. <https://doi.org/10.12029/gc20220909002>.

[9] Liu. X, Chen. Q, Zhang. Y, Gao. T, Chinese chromium demand forecasting and resource supply security. Resources Science, 2015, 37(5):37933–37943. https://www.resci.cn/CN/Y2015/V37/I5/933

[10] Wei. W, The reason of Japan’s development and stagnate in economy and its inspiration to that of China. Crossroads: Southeast Asian Studies, 2006(11):69–74. <https://kns.cnki.net/kcms2/article/abstract?v=KetWmXlLxlfLZGPGDD8RBgwCHsuEODxAvIVJ1kkVeTgpvntuA3MPrLeZ7_QZyh6pfVsI8uij5ch3qfHvN92OM2a1c7Kd4F96suDEiqbSmFVkwkFp336qvVlHqWR8zYNFrCIFzSpzs3ecfvl0FtRfp8L_NwRt&uniplatform=NZKPT>

[11] Reck B K, Rotter V S. Comparing growth rates of nickel and stainless steel use in the early 2000s. Journal of Industrial Ecology, 2012, 16(4):518–528.

1. The data is sourced from both this study and our previous research^[7]^.
